# Assessment, molecular mechanisms and therapeutic targets for renal functional reserve

**DOI:** 10.1080/0886022X.2025.2526686

**Published:** 2025-09-24

**Authors:** Zhike Fu, Yueyi Deng

**Affiliations:** Department of Nephrology, Shanghai University of Traditional Chinese Medicine, Longhua Hospital, Shanghai, China

**Keywords:** Chronic kidney disease, renal functional reserve, glomerular filtration rate, hyperfiltration

## Abstract

Chronic kidney disease (CKD) represents a significant global health challenge. Despite the availability of treatments, there remains a considerable residual risk of disease progression with current therapeutic approaches. Glomerular filtration rate (GFR) can increase due to various physiological and pathological stress responses, and the difference between the maximum GFR and the baseline GFR is termed renal functional reserve (RFR). A decline in RFR has been observed to occur well before CKD is clinically diagnosed. In addition, prolonged pathological stimulation of RFR may promote the development of other metabolic, hemodynamic, inflammatory, and fibrotic processes, which can ultimately drive CKD progression. This review consolidates the current evidence on the molecular mechanisms that underlie the initiation and decline of RFR, a phase that remains largely unaddressed as a primary treatment target but is gaining recognition for its critical role in CKD pathophysiology. Additionally, various methods for the safe and effective assessment of RFR are discussed. Recent clinical trial highlight promising new drug therapies and dietary strategies for the management of subclinical stages of CKD.

## 1. Introduction

Chronic kidney disease (CKD), which is characterized by albuminuria, low estimated glomerular filtration rate, or both, is estimated to impact more than 840 million people worldwide [1]. Glomerular filtration rate (GFR) is widely recognized as the most reliable overall indicator of kidney function [2,3]. Under resting conditions, the kidneys function at their baseline capacity, which, similar to the heart during exercise, can increase up to a certain maximum level. This ability to elevate renal function is a crucial feature that enables the kidneys to respond to physiological or pathological stimuli. The difference between the maximum GFR and baseline GFR is termed renal functional reserve (RFR) [4]. It is the ability of the kidneys to increase renal plasma flow and GFR in response to protein intake [5]. In diseased kidneys, partial activation of RFR can sustain the baseline GFR at levels that appear normal or even elevated until around 50% of functional nephrons are lost [6–8]. This suggests that RFR begins to decline before CKD is clinically diagnosed. As CKD progresses to end-stage, GFR decreases significantly, and RFR continues to decline [9]. RFR shows considerable potential for identifying subclinical signs of CKD [10], assessing kidney function recovery after acute kidney injury (AKI) [11–13], predicting adverse outcomes with medication [14], and evaluating potential kidney risks following organ transplantation [15,16].

These findings underline the importance of maintaining a higher RFR, as it quantifies the kidney’s capacity for self-regulation in response to various physiological or pathological stimuli, which is crucial for long-term renal recovery and quality of life improvement. Therefore, there is an urgent need for enhanced assessment and management of RFR, including the development of treatments that target its mechanisms, to slow CKD progression. The mechanisms behind RFR can be broadly categorized into Tubuloglomerular Feedback (TGF), blood flow autoregulation, and metabolic, endocrine, and paracrine factors. This review underscores the importance of understanding the measurement and molecular mechanisms of RFR in advancing therapeutic strategies.

## 2. Measurement of baseline GFR and RFR

### 2.1. GFR estimation by endogenous filtration markers

Accurate measurement of baseline GFR is crucial for determining RFR. The Kidney Disease: Improving Global Outcomes (KDIGO) working group reviewed evidence supporting the validity of creatinine-based GFR estimating equations and recommended the 2009 Chronic Kidney Disease Epidemiology Collaboration (CKD-EPI) creatinine equation [17] for adults and the CKiD (Children’s CKD) equation [18] for pediatric cases. Moreover, the 2021 National Kidney Foundation and American Society of Nephrology Task Force recommends using serum cystatin C to estimate GFR in adults with or at risk for CKD. Serum cystatin C is a low-molecular-weight protein present in all tissues, filtered by the glomerulus, and neither secreted into the renal tubules nor reabsorbed into the bloodstream [19,20]. These methods are cost-effective, convenient, and do not require direct urine or blood collection.

While suitable for the majority of individuals, this approach has limited external validity for certain patient populations. For instance, serum creatinine can be influenced by factors not related to kidney function. Conditions such as reduced muscle mass, lower activity levels, vegetarianism, frailty, lower extremity amputation, advanced heart failure, and liver failure are linked to creatinine production rate leading to lower serum creatinine levels [21], leading to an overestimation of GFR than the actual GFR [22]. Conversely, serum creatinine levels tend to be elevated in individuals with significant muscle mass, leading to a measured GFR that is lower than the actual GFR [19]. In contrast to serum creatinine, the factors influencing cystatin C levels remain less well understood, although they are not influenced by muscle mass or dietary intake. However, increased cystatin C concentrations have been linked to conditions such as obesity, hypothyroidism, smoking, and systemic corticosteroid administration, which may cause eGFRcys to underestimate actual GFR [17,21].

### 2.2. Direct GFR measurement by tracking the excretion rate of external markers

Postoperative renal function is largely influenced by the amount of nephron mass preserved and the level of renal functional compensation [23]. Thus, methods that directly measure GFR by tracking the excretion rate of external markers are crucial. The ideal exogenous substance for GFR measurement should be biologically inert, freely filtered through the glomerulus, readily available, and neither secreted, reabsorbed, nor metabolized in the renal tubules [17]. Existing exogenous markers of glomerular filtration are classified into three categories [24]: (1) radiopharmaceuticals, such as ^51^Cr-ethylenediaminetetraacetic acid (^51^Cr-EDTA) and ^99m^Tc-diethylenetriaminepentaacetic acid (^99m^TcDTPA), (2) nonradioactive agents like inulin, iodinated contrast media, iohexol, and unlabeled iothalamate, and (3) fluorescently labeled reagents, such as fluorescein-isothiocyanate sinistrin (FITC-sinistrin), a new GFR marker. By evaluating the rate at which these substances are filtered by the kidneys, the GFR can be calculated. GFR measured in this way is generally less impacted by confounding factors. However, each marker has specific benefits and drawbacks (Table 1), and clinicians should select the appropriate one based on individual patient circumstances.

**Table 1.** Approaches to assessing glomerular filtration rate and renal functional reserve.

methods	advantages	disadvantages
**GFR estimation by endogenous filtration markers**		
creatinine	Simple method Low cost No allergic reaction	Tubular secretion and muscle mass affect the results Not sensitive enough for measuring RFR
cystatin C	Not reabsorbed by renal tubules Simple method Low cost No allergic reaction	Can be affected by unhealthy lifestyle and hormonal imbalance Not sensitive enough for measuring RFR
**Quantifying GFR through the tracking of external marker excretion rates**		
^51^Cr-EDTA	Reliable plasma clearance	Radioactive Costly Camera methods are not Recommended Measuring RFR requires 2 days
^99m^TcDTPA λ	Reliable plasma clearance.	Radioactive Costly Accuracy affected by renal depth and injection dose Camera methods are not recommended
inulin	A ‘gold standard’ substance for measuring GFR Low toxicity Does not interfere with kidney functions during measurement	Need for infusion Measuring RFR may require a bladder catheter and multiple blood samples.
Iohexol	Reliable plasma clearance Nonionic Low cost Stable compound Easy measured Low toxicity	Requires intravenous administration More blood samples may be needed if kidney function is decreased
FITC-sinistrin	No blood sample required Reliable plasma clearance No need for anesthesia of animals during measurement Multiple continuous measurements are possible	Costly Requires intravenous administration May cause allergic reaction in human body
**Biological substances that trigger RFR**		
animal protein meal	Simple method Low cost Suitable for conscious and mildly ill patients	Non-standard response Protein content and amino acid types are not fixed
unbranched	Standard response	Costly
chain amino acids	Elimination of digestive tract effects	Requires intravenous administration Animals require respiratory anesthesia

Abbreviations: GFR, glomerular filtration rate; RFR, renal functional reserve; 51Cr-EDTA, chromium-51 ethylenediaminetetraacetic acid; 99mTc-DTPA, technetium-99m diethylenetriaminepentaacetic acid; FITC-sinistrin, fluorescein isothiocyanate–sinistrin.

### 2.3. Assessment of RFR

RFR refers to the capacity of the kidneys to increase GFR in response to physiological or metabolic stress, most commonly assessed *via* protein loading. In healthy individuals, GFR can rise following a protein intake of approximately 1.0–1.2 g/kg body weight, though the precise increase varies depending on individual factors and methodological conditions [4,25–29]. The classic formula used to calculate RFR is:

$$\text{RFR }\left(\%\right)\;=\;\left(\text{GFR}\_\text{after }-\text{GFR}\_\text{baseline}\right)/\text{GFR}\_\text{baseline }\times \text{ 1}00\%$$

[5,26]

**Figure 1.**
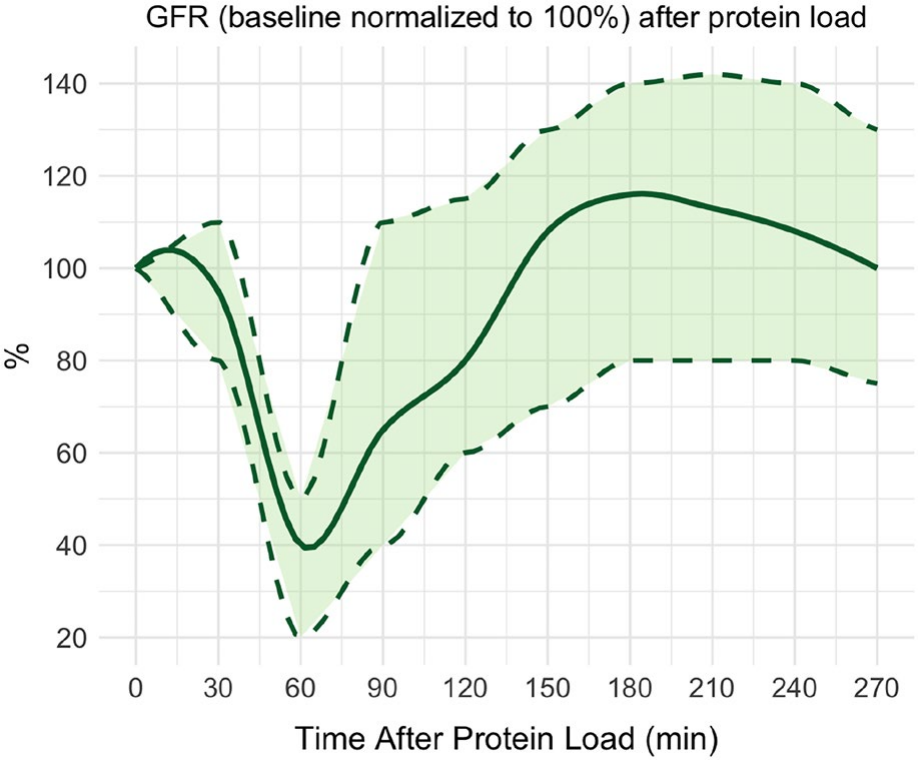
Data from Thomas Mueller et al. (2023) [29], *n* = 16, presented a new RFR protocol using ^99m^Tc-DTPA (DTPA-Cl) to assess GFR before and after an oral protein load, with the entire procedure completed in one day within an outpatient clinical setting. After a week of a low-protein diet, baseline GFR was recorded. Following a standardized hydration protocol, 1.5 g/kg body weight of beef protein was administered, and post-stimulation GFR was assessed. A 50 MBq dose of radioactivity was injected intravenously at 0 and 240 min, and plasma clearance was determined based on the activity curve at 13 time points over 480 min. RFR was defined as the difference between baseline mGFR in a fasting state and the peak mGFR after protein stimulation. The results revealed an average RFR of about 16%.

Studies have reported an average GFR increase of approximately 12%–16% in healthy individuals, corresponding to a difference of around 28.9 mL/min/1.73 m^2^ [27–29]. However, it is important to note that these values are influenced by the protein load dose, timing of measurement, measurement method (e.g., inulin vs.^99m^Tc-DTPA clearance), and individual physiology. For instance, Figure 1 shows the changes in GFR throughout the process following a 1.5 g/kg body weight dose of protein and ^99m^Tc-DTPA [29]. The results revealed an average RFR of about 16%. Radiotracers like ^99m^Tc-DTPA require up to 90 min to reach vascular-extravascular equilibrium in hydrated individuals, which can lead to transient underestimation of GFR during early post-protein-load phases [30,31].

Despite these variations, the directional response—namely, an increase in GFR following protein load—has been reliably demonstrated across studies and methods. This consistent response supports the physiological validity and reproducibility of RFR as an index of nephron adaptability. When no increase in GFR is observed following adequate protein stimulation, this may indicate a loss of renal reserve and an increased risk of underlying kidney disease.

Protein loading can be achieved through oral intake or intravenous amino acid (AA) infusion. When taken orally, animal proteins like beef are more effective at increasing GFR than plant-based proteins [32–34]. When AA are injected, unbranched chain amino acids are more efficient than branched-chain amino acids [32]. The RFR response to infusion of a complex proprietary AA mixture is more consistent [35,36]. In a multinational, double-blind trial, a dose of 2 g per kilogram of ideal body weight of AA was given over 3 consecutive days to adult patients undergoing cardiac surgery, a high-risk group for AKI. The risk of adverse events did not increase in the AA group [35].

Notably, body weight normalization is a key issue. Some studies use actual body weight, while others use ideal body weight, calculated *via* formulae such as those by Boer, James, or Hume. Given that fat-free mass more closely reflects metabolic demand and glomerular filtration capacity, the use of ideal body weight is considered more appropriate in many settings.

Animal studies using approved amino acid infusions (e.g., Synthamin^®^ 17) have shown that RFR recruitment leads to increased oxygen tension (PO_2_) in both cortical and medullary renal tissues [37]. While this may indicate improved perfusion, the possibility of deleterious hyperperfusion in specific pathological settings must also be acknowledged [38]. Hence, the context and duration of RFR recruitment are key considerations in interpreting its effects.

It is important to recognize that absolute RFR values may differ among protocols, but within-study comparisons using standardized methods remain robust and informative. While methodological heterogeneity currently limits direct numerical comparisons between studies, this does not undermine the overall utility of RFR assessment. On the contrary, early identification of impaired RFR may offer a window of opportunity for intervention before irreversible renal injury occurs.

In conclusion, current methods enable the safe and reproducible measurement of RFR in both experimental and clinical settings. However, due to methodological heterogeneity across studies—such as differences in protein load dosage, measurement timing, and normalization strategies—direct numerical comparisons of absolute RFR values between different studies or clinical environments are not currently reliable. Instead, within-subject comparisons using consistent and standardized protocols remain the most appropriate approach to assess changes in RFR. Moving forward, the development and adoption of harmonized protocols—including standardized protein load dosing, measurement intervals, and normalization methods—will be essential to improve inter-study comparability and enhance the clinical utility of RFR assessment. (See Table 1 for a summary of methodological features and considerations.)

## 3. Mechanisms that stimulate RFR

**Figure 2.**
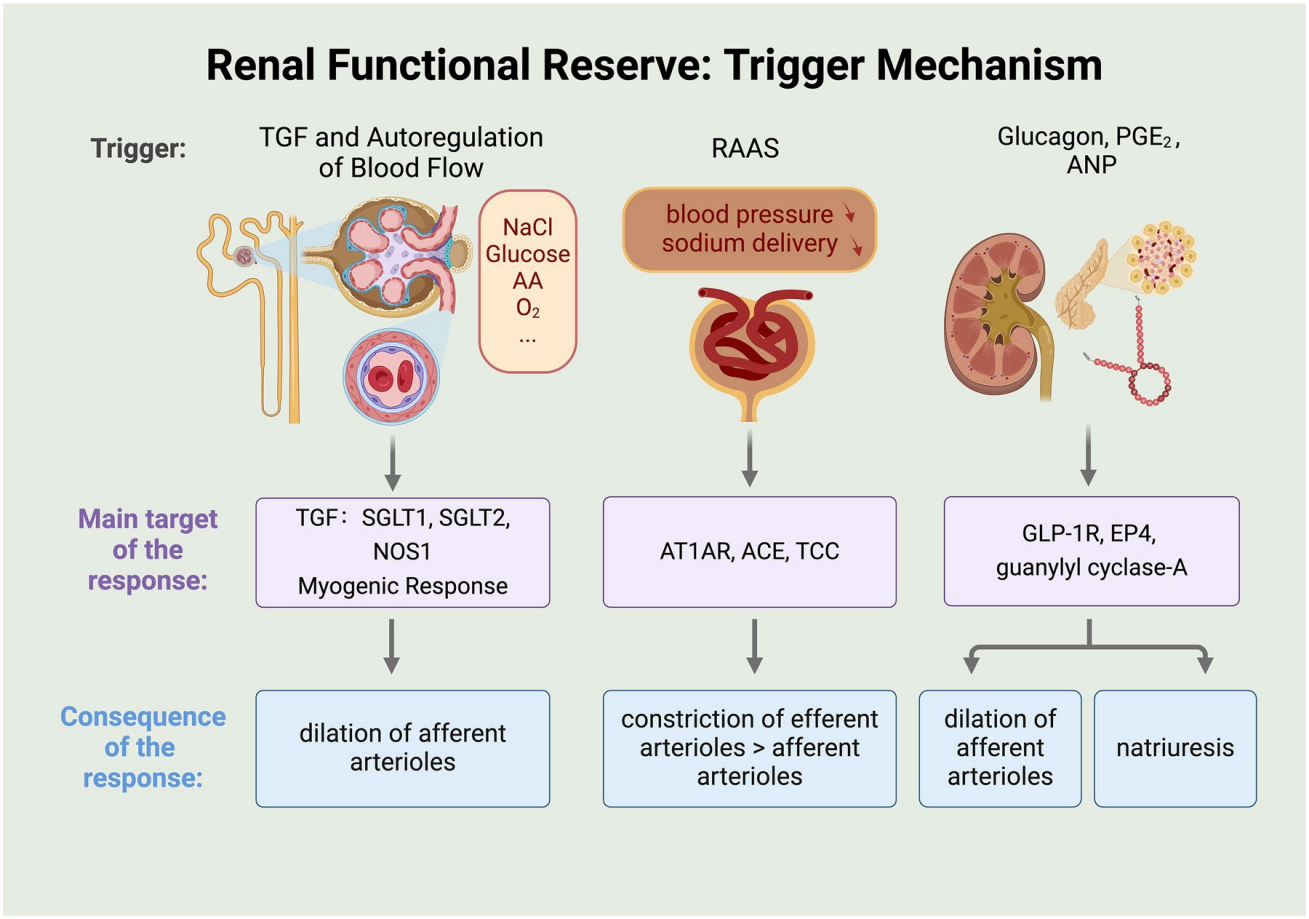
Proposed mechanisms of renal functional reserve following protein loading or amino acid infusion. Initially, sodium-glucose cotransporters 2 (SGLT2) and 1 (SGLT1) reduce sodium chloride (NaCl) delivery to the macula densa, which triggers relaxation of the afferent arteriole and enhances the glomerular filtration rate (GFR) of the nephrovascular unit (NVU). The myogenic response of the renal vascular tree then allows the NVUs to adjust their afferent arteriolar tone in response to pressure fluctuations. Subsequently, angiotensin II (ANG II) causes more pronounced constriction of efferent arterioles compared to afferent arterioles *via* the ANG II type 1 A receptor (AT1AR) and angiotensin-converting enzyme (ACE). This action is mediated by the activation of T-type Ca^2+^ channels (TCCs), facilitating Ca^2+^ entry into blood vessels. Lastly, certain autocrine and paracrine factors, such as glucagon, prostaglandin E2 (PGE_2_), and atrial natriuretic peptide (ANP), influence glucagon-like peptide receptors (GLP-1R), various G protein-linked receptors (EP_4_), and guanylyl cyclase-A, resulting in afferent arteriole dilation and enhanced urinary natriuresis, which together increase GFR.

Protein loading causes alterations in renal hemodynamics [5,39,40]. The extent of GFR changes, or RFR, directly reflects these renal hemodynamic shifts.25 What factors contribute to the activation of RFR in response to different physiological stimuli? (shown in Figure 2)

### 3.1. Tubuloglomerular Feedback (TGF)

A considerable body of evidence supports the essential role of TGF in the elevation of GFR following systemic administration of amino acids [41,42]. Specialized cells in the juxtaglomerular apparatus, known as macula densa cells, detect changes in sodium chloride concentrations in the tubular fluid. When sodium chloride levels drop, these cells signal the afferent arterioles to dilate, thus increasing GFR to maintain proper filtration [6]. Two main pathways linked to TGF have been proposed in the literature.

#### 3.1.1. SGLT2-NaCl-TGF pathway

Some studies suggest that tubular growth and the upregulation of sodium-glucose cotransporter 2 (SGLT2) in diabetic conditions enhance proximal tubular reabsorption, decreasing NaCl delivery to the macula densa and, in turn, increasing GFR *via* TGF [43]. SGLT2 inhibitors allow for more sodium to pass along the nephron, which is sensed by macula densa cells. These cells, in turn, signal *via* adenosine to constrict afferent glomerular arterioles, reducing intraglomerular pressure and providing protection to the glomerulus. The cumulative effects of SGLT2 inhibitors may improve tubular oxygenation and metabolism, while also reducing renal inflammation and fibrosis. SGLT2 inhibitors do not appear to increase the risk of urinary tract infections or acute kidney injury [44]. However, due to the initial decrease in GFR, the introduction of SGLT2 inhibitors in patients with very low GFR is not recommended, and treatment should be stopped with caution in cases of acute renal events, hypovolemia, or hypotension [45].

#### 3.1.2. SGLT1 -NOS1-TGF pathway

Recent research has also highlighted the role of sodium-glucose cotransporter 1 (SGLT1) in TGF [46,47]. Zhang Jie et al. showed that knocking out NOS1 specifically in the macula densa reduced the suppression of TGF and GFR elevation induced by diabetes. Additionally, deleting SGLT1 in macula densa cells prevented the upregulation of NOS1 in these cells, mitigating the suppression of TGF in diabetic mice [47]. They also demonstrated that SGLT1 activation promotes the production of nitric oxide (NO), contributing to glomerular hyperfiltration [48,49]. Although ultrafiltration restores sodium and chloride excretion, it places additional stress on the filtration barrier and increases oxygen demand for reabsorption [49].

### 3.2. Autoregulation of blood flow

GFR can fluctuate within a certain range in response to various physiological or external stimuli, a process largely maintained by the kidney’s unique ability to autoregulate blood flow [50]. This autoregulatory capacity is primarily mediated by a combination of TGF mechanisms and the generic myogenic response [51].

Although RFR is typically quantified by observing changes in GFR following physiological or pharmacological challenges, the underlying autoregulatory mechanisms help explain how the kidney adjusts filtration capacity in response to increased demand.

For effective autoregulation, nephrons communicate electrically over long distances with other nephrons through the vascular tree, rather than functioning as individual units to manage blood flow [51,52]. The renal vascular tree exhibits irregular topology, leading to variations in preglomerular pressure drops across nephrovascular units (NVUs), with these differences becoming more apparent during autoregulation’s response to blood pressure fluctuations [53]. NVUs with greater or lesser preglomerular pressure drops can adjust their afferent arteriolar tone in response to pressure changes.

TGF operates within each individual NVU. Increased NaCl reabsorption at the macula densa triggers the release of ATP and adenosine, which diffuse into the extraglomerular mesangium where they activate P2X (ATP) and A1 (adenosine) receptor signaling [54]. Communication between NVUs involves two essential components. One is the vasoconductive response (VCR), a depolarization initiated by ATP and adenosine across the glomerular mesangium to the afferent arterioles, leading to an upstream vasomotor response [55]. The second component is connexins (CXs), where gap junctions formed by transmembrane CX proteins create axial and radial communication pathways, allowing TGF signaling to be transmitted through electrical currents [56]. These signals are crucial for triggering the generic myogenic response.

It is important to note, however, that due to significant regional heterogeneity in glomerular perfusion, imaging-based assessments of renal blood flow (e.g., Doppler ultrasound) may not accurately reflect RFR and should be interpreted with caution.

### 3.3. Activation of the renin-angiotensin-aldosterone system (RAAS)

In contrast to earlier findings, the renin-angiotensin system (RAAS) appears to be significantly involved in renal hemodynamic changes after protein loading. Typically, when blood pressure drops or there is a decrease in sodium delivery to the distal tubules, the RAAS is triggered. This process leads to the release of renin, which eventually results in the production of angiotensin II (ANG II). The role of ANG II is to activate T-type Ca^2+^ channels (TCC), facilitating Ca^2+^ entry into blood vessels [57]. This causes the constriction of efferent arterioles and increases sodium reabsorption in the proximal renal tubule, helping to preserve GFR under low blood pressure conditions [58]. In individuals with hypertension, RFR is lower than in healthy individuals despite near-normal renal function and is associated with specific blood pressure levels [5]. Additionally, these individuals show an inadequate response to protein loading while taking antihypertensive medications such as ACEIs and ARBs [5].

Recent evidence indicates that TGF responses are absent in mice lacking the ANG II type 1 A (AT1A) receptor [59] or those without endothelial angiotensin-converting enzyme (ACE) [60]. Furthermore, ACE2 KO mice do not exhibit hyperfiltration on a high-protein diet [61], suggesting that changes in ACE2 activity or expression may be essential for restoring RFR following protein loading.

### 3.4. Glucagon release

When amino acids were administered to humans and animals, glucagon levels were observed to rise only with increased amino acid concentrations [62,63]. In both humans and animals, the injection of supraphysiological doses of glucagon [64,65] or glucagon mixed with other hormones [66] resulted in an increase in renal plasma flow (RPF) and GFR [67]. Additionally, in pancreatectomy patients receiving a mixed amino acid solution, there was no observed increase in RPF or GFR when compared with normal subjects [68]. These results provide some evidence that glucagon plays a role in increasing GFR during amino acid loading.

Recently, glucagon-like peptide-1 receptors (GLP-1R) have been identified in the renal vasculature, including afferent arterioles [69], where they exert a direct vasodilatory effect [70,71]. Moreover, GLP-1R is widely present in renal tubules [72] and promotes natriuresis by inhibiting sodium-hydrogen exchanger isoform 3 (NHE3) in the proximal tubule [73,74]. As a result, GLP-1 antagonizes the action of SGLT2.

### 3.5. Prostaglandin release

Prostaglandins, particularly prostaglandin E2 (PGE_2_), the predominant prostaglandins produced by the kidneys, act as vasodilators and are released in response to various stimuli, such as increased blood pressure or reduced sodium delivery [75]. 6-keto-PGF_1α_, a stable metabolite of prostacyclin, is also involved in these processes. GFR and the synthesis rates of PGE_2_ and 6-keto-PGF_1α_ in the glomeruli were significantly elevated after oral protein loading in rats [76]. Furthermore, indomethacin blocked the glucagon-induced increases in RPF and GFR [77], indicating that prostaglandin compounds are essential for amino acid loading to stimulate glucagon release and thus enhance RPF and GFR [78]. PGE_2_ promotes the dilation of afferent arterioles [77,79], thereby increasing blood flow to the glomeruli.

However, other studies have indicated that PGE_2_ may also cause afferent arteriolar constriction [79], suggesting the involvement of multiple G protein-coupled (EP) receptors. At lower concentrations, PGE_2_ activates EP_4_ receptors, stimulating adenylyl cyclase and increasing cAMP production. At higher concentrations, or when EP_3_ receptor expression is upregulated, PGE_2_ continues to stimulate EP_3_ receptors, counteracting the effects of EP_4_ and promoting vasoconstriction by reversing the vasodilation caused by PGE2 [79].

### 3.6. Release of natriuretic peptides

Natriuretic peptides, such as atrial natriuretic peptide (ANP) and brain natriuretic peptide (BNP), are released in response to increased blood volume and pressure. Studies have shown that signaling through natriuretic peptides, guanylyl cyclase-A, and cyclic guanosine monophosphate (cGMP) can significantly reduce aldosterone-induced renal damage in mice [80]. These peptides promote vasodilation of afferent arterioles [81] and inhibit sodium reabsorption in renal tubules [82], thereby helping to increase GFR [83]. A 1990 study suggested that ANP may not be directly involved in the hemodynamic response following protein loading, though it did not rule out an indirect role [84]. In contrast, a 1994 study highlighted the significant role of ANP in the increase in GFR observed after amino acid infusion. Researchers found altered plasma levels of ANP and glucagon following amino acid injections in both healthy subjects and patients with chronic glomerulonephritis [85]. The increase in ANP may be related to plasma volume expansion, resulting from the binding of water and amino acids.

## 4. Mechanism of RFR loss

### 4.1. Renal vascular damage

RFR is susceptible to a range of pathological insults. Experimental studies have demonstrated that RFR progressively declines following ischemia-reperfusion injury in rat models [12]. Interestingly, this reduction can be mitigated by hydrodynamic delivery of isotonic fluids [86]. This intervention, particularly with saline, has been shown to alleviate vascular congestion, reduce Th17 cell infiltration, and accelerate early vascular recovery post-ischemia [87]. These findings suggest that renal vascular damage may be a key factor in reduced RFR in AKI.

In the context of subclinical CKD, persistent stimuli that elevate glomerular pressure—such as ANG II—can induce the expression of pro-inflammatory and pro-fibrotic mediators. This occurs through both mechanical stress and direct cellular effects [88]. As a result, impaired renovascular reactivity and diminished microvascular density are thought to be critical factors underlying the loss of RFR [89].

### 4.2. Renal tubular dysfunction

Beyond vascular injury, renal tubular damage also plays a pivotal role in the decline of RFR. Changes in specific urinary biomarkers have been observed even when the GFR remains stable. For instance, elevated urinary levels of tissue inhibitor of metalloproteinases-2 (TIMP-2) and insulin-like growth factor-binding protein 7 (IGFBP7) have been associated with early RFR loss [13]. These biomarkers are linked to G1 cell cycle arrest, an early response to tubular cell stress [90].

Tubular dysfunction also disrupts tubuloglomerular balance. In hyperglycemic conditions, for example, the expression of SGLT2 is upregulated on the luminal surface of proximal tubular epithelial cells. This impairs TGF and leads to glomerular hyperfiltration, a state that compromises glomerular hemodynamics and diminishes RFR [91].

### 4.3. Hyperfiltration

Certain pathological states may cause an elevation in baseline GFR prior to any physiological challenge, resulting in a seemingly reduced RFR. In early diabetic nephropathy, hyperfiltration is commonly observed, characterized by an abnormally high GFR before overt kidney damage occurs. This elevated baseline GFR reduces the apparent increase in GFR following stimulation, thereby masking the true RFR [92,93].

Taken together, these mechanisms—including vascular injury, tubular stress, disrupted feedback systems, and elevated baseline filtration—illustrate the multifactorial nature of RFR loss and underscore its relevance in early renal dysfunction.

## 5. Therapeutic interventions in renal functional reserve

RFR measurement is commonly used to predict kidney disease progression. For instance, a decline in RFR is seen early in renal involvement in systemic sclerosis [94]; a decrease in RFR also occurs with the onset of low-grade albuminuria in early diabetic nephropathy [95]; and a decline in RFR precedes increases in serum creatinine and GFR decline in subclinical CKD following renal injury [12]. Can interventions effectively address the decline in RFR before the diagnosis of kidney disease? Could stimulating RFR represent a viable treatment strategy for kidney disease?

### 5.1. Blood pressure control

In hypertensive patients, the disruption of renal vasodilatory mechanisms compromises the kidney’s ability to increase GFR in response to physiological stimuli, resulting in a reduced RFR [96]. RFR, as a reflection of the kidney’s adaptive reserve, has been shown to be significantly lower in hypertensive individuals, even when baseline GFR remains within normal limits [5].

In a clinical study, patients with essential hypertension showed a blunted RFR response to protein loading, indicating impaired renal vascular adaptability [97]. antihypertensive therapy with carvedilol partially restored RFR, suggesting that blood pressure control may help preserve renal reserve [97]. These findings support the use of RFR as a marker of renal vascular integrity and underscore the importance of early blood pressure control to prevent progression to overt renal dysfunction.

### 5.2. Diabetes management

In early diabetes, glomerular hyperfiltration and impaired TGF sensitivity lead to reductions in RFR, even in the presence of a normal eGFR [98]. Additionally, in diabetic patients, reductions in RFR often precede or coincides with low-grade albuminuria and are considered early signs of diabetic nephropathy [99,100]. Therefore, early detection of reduced RFR in diabetes should prompt adjustments in glycemic control strategies to help prevent further declines in GFR.

#### 5.2.1. SGLT2 inhibitors

SGLT2 inhibitors reduce hyperfiltration and restore TGF sensitivity. In an 8-week study, empagliflozin reduced hyperfiltration and increased afferent arteriolar resistance in patients with T1DM, suggesting restoration of renal autoregulation and potential improvement in RFR [101,102]. In longer-term trials in T2DM, initial reductions in eGFR with SGLT2 inhibitors are hemodynamic and reversible, aligning with improved RFR dynamics [103].

#### 5.2.2. GLP-1 receptor agonists

GLP-1 receptor agonists such as liraglutide reversibly lower GFR *via* sodium reabsorption inhibition and TGF activation [104]. This effect modulates glomerular pressure and may preserve RFR by restoring physiological feedback mechanisms [105].

#### 5.2.3. Thiazolidinediones

Pioglitazone has been shown to attenuate glomerular hyperfiltration and improve renal vascular response in diabetic models [106,107]. Its action on PPAR-γ and Klotho pathways may indirectly preserve RFR by reducing ANG II–induced glomerular injury and maintaining hemodynamic reserve [106].

### 5.3. BMI management

Excess body weight, particularly obesity, has been associated with reductions in RFR. For example, obese hypertensive patients demonstrate significantly lower RFR than lean hypertensive individuals, indicating compromised renal adaptability despite elevated baseline GFR [108]. Encouragingly, weight loss has been shown to reverse obesity-induced hyperfiltration and may help preserve RFR [109].

BMI is positively correlated with GFR, which can be influenced either directly through adipose tissue hypertrophy and ectopic fat accumulation in the kidneys, or indirectly *via* increased risk of hypertension and diabetes [110] Evidence from kidney donor studies also supports this relationship: higher BMI is associated with a greater decline in RFR after nephrectomy [16].

At the pathophysiological level, abnormal lipid accumulation in the kidneys leads to glomerular hypertension [111], hyperfiltration, glomerular enlargement, and proteinuria [109,112].

Emerging therapeutic strategies targeting renal lipid metabolism may help mitigate these effects. For instance: Farnesoid X receptor (FXR) agonists have been shown to reduce renal lipid deposition by inhibiting sterol regulatory element binding protein-1 (SREBP-1) [113,114]. Peroxisome proliferator-activated receptor alpha (PPARα) agonists, such as fenofibrate, limit fatty acid and triglyceride accumulation and reduce proteinuria progression, albeit with a transient decrease in GFR [115]. TGR5 (G protein-coupled bile acid receptor 1) agonists improve mitochondrial β-oxidation of fatty acids and reduce renal lipid accumulation in obesity models [116].

Overall, interventions targeting weight reduction and renal lipid metabolism appear to be beneficial in protecting or restoring RFR, particularly in patients with obesity-related glomerular stress.

### 5.4. Protein intake

Protein intake has a complex relationship with RFR. On one hand, high-protein diets can induce glomerular hyperfiltration, which may lead to a progressive decline in kidney function and a reduction in RFR. On the other hand, controlled short-term protein loading may offer potential renal protection in specific clinical scenarios.

For example, In a multinational, double-blind trial, adult patients undergoing cardiac surgery with cardiopulmonary bypass were randomly assigned to receive either an intravenous infusion of a balanced amino acid mixture (2 g per kilogram of ideal body weight per day) or a placebo (Ringer’s solution) for up to 3 days. AKI occurred in 474 patients (26.9%) in the amino acid group and 555 patients (31.7%) in the placebo group (relative risk, 0.85; 95% confidence interval [CI], 0.77 to 0.94; *p* = 0.002). There were no significant differences between the groups in other secondary outcomes or adverse events [35]. This suggests that short-term, high-dose protein administration may not be harmful and could have protective effects in acute settings.

In healthy adults with prehypertension—a condition where blood pressure levels range between normal and hypertensive thresholds (typically 120–139 mmHg systolic or 80–89 mmHg diastolic) [117]—a high-protein diet was associated with an increase in eGFR as measured by cystatin C. Specifically, a 4 mL/min/1.73 m^2^ increase in cystatin C-based eGFR was observed compared to those on a diet high in carbohydrates and unsaturated fats [118].

Nevertheless, sustained hyperfiltration may contribute to progressive kidney damage over time. In a longitudinal study of 2,255 patients with prior myocardial infarction, higher protein intake was associated with a faster decline in kidney function over a 41-month follow-up [119]. Notably, this negative effect may be attenuated by the use of SGLT2 inhibitors, which have been shown to counteract glomerular hyperfiltration [120,121].

In summary, while short-term or moderate protein intake within recommended dietary limits may be safe or even beneficial in certain populations, long-term excessive protein consumption is generally discouraged in individuals at risk of kidney disease. Avoiding hyperfiltration through dietary moderation may help preserve RFR and slow the progression of renal impairment.

## 6. Conclusion

RFR has great potential for identifying subclinical stages of CKD, monitoring renal function recovery following AKI, predicting adverse outcomes during treatment, and assessing potential kidney risks after organ transplantation.

There are various methods for measuring RFR, and it is important for clinicians and researchers to choose the most appropriate method depending on the specific study population. The recommended protein load dose for achieving the maximum GFR while minimizing the risk of side effects is typically between 1 and 1.2 g of protein per kilogram of body weight.

Stimulating RFR mainly involves changes in glomerular hemodynamics. This process is driven by mechanisms such as TGF, renal blood flow autoregulation, RAAS activation, and the release of glucagon, prostaglandins, and natriuretic peptides. These factors cause afferent arterioles to dilate and/or efferent arterioles to constrict, thereby increasing glomerular pressure and GFR. As a result, RFR can be quantified. This response is temporary and self-regulated, with no serious side effects reported. However, prolonged glomerular hyperfiltration can lead to the activation of pro-inflammatory and pro-fibrotic mediators, resulting in RFR decline.

Several therapeutic targets, including SGLT2, SGLT1, GLP-1R, and potentially selective inhibitors to reduce glomerular hypertension, are being explored to further reduce risks and enable more tailored therapies. Additionally, basic dietary approaches such as avoiding high-protein and managing BMI can also help prevent RFR loss.
